# Host Cell Restriction Factors that Limit Influenza A Infection

**DOI:** 10.3390/v9120376

**Published:** 2017-12-07

**Authors:** Fernando Villalón-Letelier, Andrew G. Brooks, Philippa M. Saunders, Sarah L. Londrigan, Patrick C. Reading

**Affiliations:** 1 Department of Microbiology and Immunology, The University of Melbourne at the Peter Doherty Institute for Infection and Immunity, Melbourne, VIC 3000, Australia; fvillalon@student.unimelb.edu.au (F.V.-L.); agbrooks@unimelb.edu.au (A.G.B.); philippa.saunders@unimelb.edu.au (P.M.S.); sarahll@unimelb.edu.au (S.L.L.); 2 WHO Collaborating Centre for Reference and Research on Influenza, Victorian Infectious Diseases Reference Laboratory at the Peter Doherty Institute for Infection and Immunity, Melbourne, VIC 3000, Australia

**Keywords:** influenza, innate, restriction factor, replication, interferon-stimulated gene

## Abstract

Viral infection of different cell types induces a unique spectrum of host defence genes, including interferon-stimulated genes (ISGs) and genes encoding other proteins with antiviral potential. Although hundreds of ISGs have been described, the vast majority have not been functionally characterised. Cellular proteins with putative antiviral activity (hereafter referred to as “restriction factors”) can target various steps in the virus life-cycle. In the context of influenza virus infection, restriction factors have been described that target virus entry, genomic replication, translation and virus release. Genome wide analyses, in combination with ectopic overexpression and/or gene silencing studies, have accelerated the identification of restriction factors that are active against influenza and other viruses, as well as providing important insights regarding mechanisms of antiviral activity. Herein, we review current knowledge regarding restriction factors that mediate anti-influenza virus activity and consider the viral countermeasures that are known to limit their impact. Moreover, we consider the strengths and limitations of experimental approaches to study restriction factors, discrepancies between in vitro and in vivo studies, and the potential to exploit restriction factors to limit disease caused by influenza and other respiratory viruses.

## 1. Influenza Virus

Within the Orthomyxoviridae family, influenza viruses are commonly associated with respiratory disease. Influenza viruses contain a segmented genome of single-stranded negative sense RNA and can be divided into types A, B, C and D based on antigenic differences in the internal nucleoprotein (NP) and matrix (M) proteins. Type A, B and C viruses readily infect humans, with type A and B influenza viruses (IAV and IBV, respectively) commonly associated with respiratory disease. IAV can be further divided into subtypes depending on the genetic and antigenic properties of the hemagglutinin (HA) and neuraminidase (NA) surface glycoproteins. To date, 18 different HAs and 11 different NAs have been identified, with most isolated from aquatic birds, although some that have also been identified in bats and other mammals [1]. In the last 100 years, only the H1N1, H2N2 and H3N2 subtypes have been associated with pandemics [2]. Currently, IAV of the H1N1pdm09 and H3N2 subtypes, as well as IBV, circulate in humans and are responsible for seasonal (or epidemic) influenza.

Vaccination and antiviral drug treatment represent the most common strategies used to limit the impact of influenza in the human population. Vaccination represents the most effective method for prevention and control, reducing illness as well as disease severity in high-risk groups, including young children and the elderly (reviewed in [3]). Influenza vaccines require annual evaluation and reformulation in order to match the most recently circulating strains, which continually change as a result of antigenic drift [4]. Inhibitors of the viral M2 ion channel (adamantanes) or the neuraminidase (neuraminidase inhibitors or NAI) represent antiviral drugs approved for use in humans, although most circulating human IAV are now resistant to adamantanes [5]. NAI are widely used, although their usefulness in treating seasonal influenza and their need to be stockpiled for use in a future influenza pandemic have been the subject of much debate [6].

## 2. IAV Infection of Host Cells

Airway epithelial cells (AEC) represent the primary targets of influenza virus infection, although immune cells such as airway macrophages (AMΦ) and dendritic cells (DC) are also susceptible. While infection of AEC by seasonal influenza viruses results in productive replication and amplification of virus, this process is generally considered abortive in AMΦ and DC and infectious progeny are not released (reviewed in [7]). Irrespective of cell type, influenza virus infection is initiated following attachment of the viral HA glycoprotein to terminal sialic acid (SIA) expressed by glycoproteins and glycolipids on the surface of target cells. Following virus attachment, entry generally occurs via receptor-mediated endocytosis, although the specific entry receptors for influenza virus remain poorly defined, particularly for AEC. Recent work in our laboratory has identified C-type lectin receptors as attachment and entry receptors for influenza virus infection of MΦ (reviewed in [8]). Influenza virus enters cells by a range of different pathways, including clathrin- or caveolae-mediated endocytosis, as well as macropinocytosis [9] and this appears to vary depending on the cell types, viral strains and culture conditions utilised. After internalization, viruses are trafficked to late endosomes where the viral HA is activated following induction of a pH-dependent conformational change, exposing the fusion peptide, which binds to the endosomal membrane to facilitate fusion. The interior of the virus particle is also acidified by the M2 ion channel, allowing the depolymerization of the M1 and subsequent dissociation from the viral ribonucleoproteins (vRNPs). As a result, vRNPs are released through the fusion pore into the cytosol and then imported into the nucleus, where the transcription and replication occurs [10]. Following replication of the viral genome, synthesis of viral mRNAs and translation of at least 10 viral proteins, new virions assemble at the cell surface. The HA and NA glycoproteins are targeted to lipids rafts, where they can cause deformation of the plasma membrane to initiate virus budding. Finally, the viral NA acts to cleave SIA, preventing virus aggregation at the cell surface and promoting release of newly-synthesised virions.

## 3. Induction of Type I Interferons and Innate Immunity Following IAV Infection

**Figure 1:**
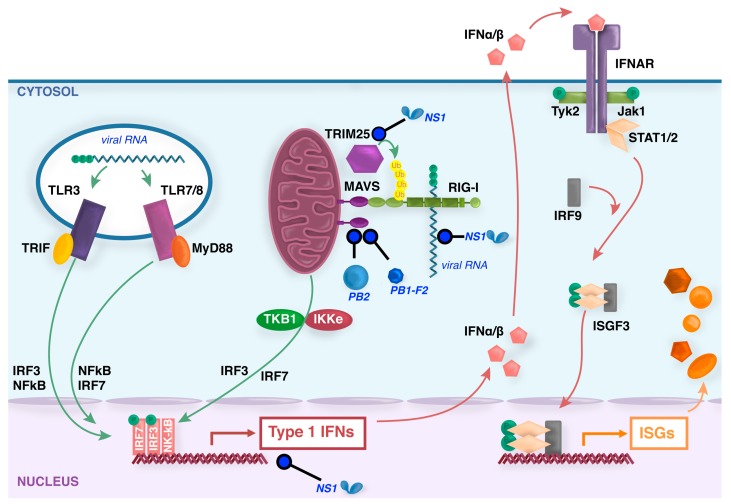
Induction of type I interferons (IFNs) by influenza viruses. During entry of influenza virus into some host cells, virions within the endosome expose viral RNA to toll-like recepotrs (TLR)3/7/8. Virus replication produces triphosphorylated vRNA and potentially dsRNA by-products that are recognized by the ubiquitously expressed cytoplasmic sensor retinoic acid inducible gene (RIG)-I. TLR signaling by the adaptors TIR-domain-containing adapter-inducing interferon-β (TRIF) or myeloid differentiation factor (MyD)88, and RIG-I signaling by the adaptor mitochondrial antiviral signaling (MAVS), trigger signal transduction cascades that result in activation of IFN regulatory factor (IRF)3/7 and NF-κB factors that translocate to the nucleus to induce synthesis of type I IFN mRNAs. Secreted type I IFNs signal through the IFN-α/β receptor complex (IFNAR) via activation of the intracellular kinases Jak1 and Tyk2, which phosphorylate the signal transducer and activator of transcription (STAT) transcription factors that, together with IRF-9, form the interferon-stimulated gene factor 3 (ISGF3) that translocates to the nucleus and activates the transcription of ISGs. In virus-infected cells, the nonstructural (NS)1 protein binds to and sequesters dsRNA to antagonize RIG-I activation, as well as interacting with various host proteins to inhibit transcription, processing or nuclear export of cellular mRNAs [20,21]. NS1 also binds to tripartite motif-containing (TRIM)25 protein and prevents essential ubiquitination of RIG-I [22]. Viral PB1-F2 [23] and PB2 proteins [24,25] inhibit MAVS function in the mitochondria.

It is well established that infection of host cells by viruses, including influenza viruses, induces secretion of type I interferons (IFN). Induction of type I IFNs is dependent on a range of cellular pattern recognition receptors (PRRs) that recognise pathogen-associated molecular patterns (PAMPs) expressed by the virus and/or generated during the viral infection. Depending of the nature of the virus and/or the target cell, different pathways can be activated to induce type I IFNs. RNA viruses such as influenza virus can be detected by at least two different classes of PRRs: Toll-like receptors (TLR) and retinoic acid inducible gene (RIG)-I-like receptors (RLR) (reviewed in [11]). TLR3 is expressed in endosomal compartments of different cell types relevant to influenza virus infection, including AEC and DC. Human TLR8 and mouse TLR7, also expressed within endosomes, can sense ssRNA to promote the transcription of IFNs (Figure 1). In addition to endosomal PRRs, most cells in the body express cytosolic PRRs of the RLR family, including RIG-I and melanoma differentiation associated gene (MDA)-5, which recognize cytoplasmic viral RNA. RIG-I recognizes short RNA ligands with 5′-triphosphate (5′-ppp) caps that are generated during viral replication while MDA-5 tends to recognize long kilobase-scale genomic viral RNA and replication intermediates (reviewed in [12]) (Figure 1). It is well established that IFN induction in response to IAV in AEC and other cell types is predominantly RIG-I-dependent [13,14,15]. However, studies suggest that MDA-5 may play a supportive role in contributing to IFN induction as MDA5-deficient cells do show reduced IFN induction after exposure to IAV [14,15,16]. Following intracellular sensing by PRRs, type I IFNs secreted from virus-infected cells bind to the ubiquitously expressed interferon α/β receptor (IFNAR), inducing signaling pathways and resulting in the transcription of hundreds of IFN-stimulated genes (ISGs). This results in induction of an “antiviral state” in cells, which limits further virus replication and spread (reviewed in [17]). Several recent reviews have addressed the induction of type I IFNs and their role during influenza virus infections [18,19]. Moreover, influenza viruses have evolved diverse strategies to minimize IFN expression and/or signaling. In particular, the viral NS1 protein acts to antagonise the host IFN response through several distinct molecular mechanisms (reviewed in [20]). Figure 1 shows induction of type I IFNs and countermeasures that influenza viruses use to limit IFN induction.

## 4. Induction of Cellular Restriction Factors: ISGs and Other Antiviral Factors

It is well known that type I IFNs are potent mediators of antiviral activity against a broad range of viruses, however there is still limited knowledge regarding the specific effector molecules involved and where they act in the life cycle of different viruses. At the transcriptional level, viral infections have been reported to induce hundreds of ISGs and there is strong evidence that some ISGs are critical effectors against particular viruses (reviewed in [26]). However, of the many ISGs reported, functional and biochemical analyses of only a small subset of ISG proteins have been described. Amongst the well-studied ISGs with antiviral effects against a broad range of viruses are the myxovirus resistance (Mx) family of guanosine triphosphate (GTP)ases, the 2′-5′ oligoadenylate synthetase (OAS)-directed RNaseL pathway family proteins, protein kinase R (PKR) and members of the interferon-inducible transmembrane (IFITM) family (reviewed in [27]). While some ISG proteins exhibit broad antiviral activity, others appear to be specific for one or more virus families. Moreover, there are still many ISGs that have not been assessed for antiviral activity against different virus families. In addition to ISGs, host cells express a range of other proteins with antiviral activity that are regulated independently of type I IFNs [28]. ISGs and other cellular proteins with antiviral activity (hereafter referred to as “restriction factors”) may be expressed constitutively at different levels in particular cell types and levels may be modulated by virus infection, type I IFNs and/or other factors.

**Figure 2:**
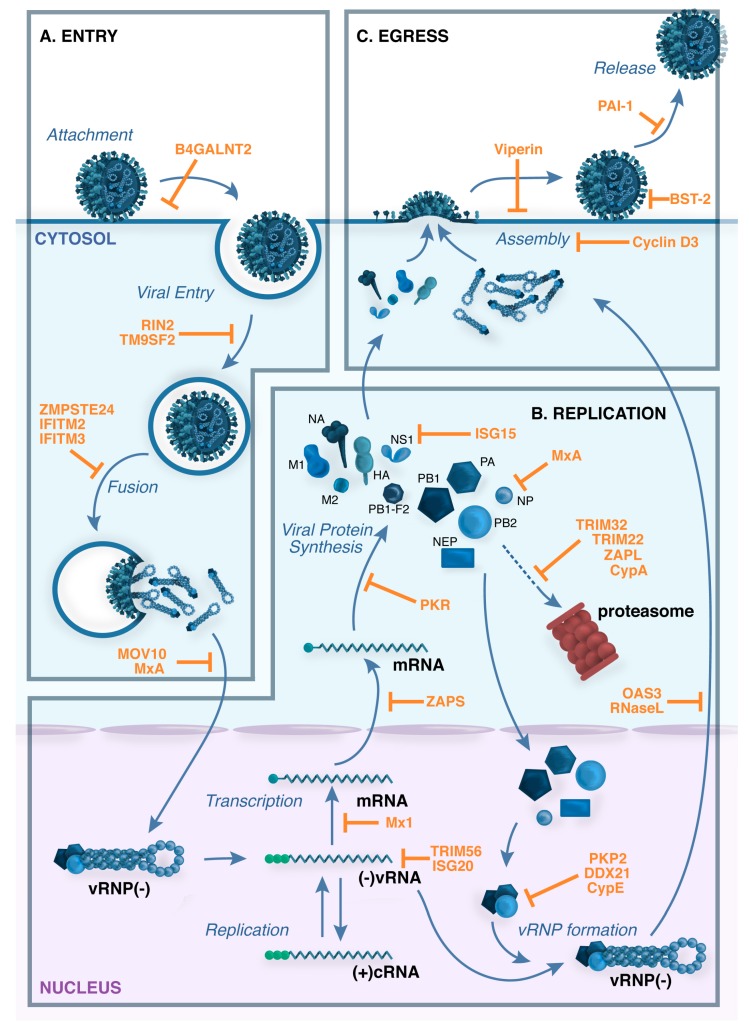
Restriction factors block multiple stages in the influenza virus life cycle. (**A**) Virus attachment and entry. The influenza virus hemagglutinin (HA) attaches to host cell receptors that contain terminal α2,6-linked or α2-3-linked sialic acid moieties, and the virus enters the cell by receptor-mediated endocytosis. Cleavage of HA by cellular proteases is required to expose the HA peptide that is responsible for the fusion between the viral envelope and the endosomal membrane. Acidification of the endocytic vesicle opens the M2 ion channel, resulting in acidification of the inside of the virion, a process that is required for proper uncoating of the viral ribonucleoprotein (vRNP) complexes that contain the viral genome. Acidification of the endosome also triggers the pH-dependent fusion step that is mediated by the HA and results in the cytoplasmic release of the vRNP complexes; (**B**) Genomic transcription and replication. vRNPs translocate to the nucleus, where the RNA-dependent RNA polymerase transcribes and replicates the negative-sense viral RNA ((−)vRNA), giving rise to: (i) complementary positive-sense RNA ((+)cRNA), which is used as a template to generate more vRNA; and (ii) viral mRNAs, which are exported to the cytoplasm for translation; (**C**) Virus assembly and budding. Viral proteins that are needed in replication and transcription are translocated back to the nucleus, and progeny vRNPs are then exported to the cytoplasm for packaging. Viral HA, NA and M2 are transported by the trans-Golgi secretory pathway to the plasma membrane, where M1 assists in the formation of mature virus particles. Virions then bud from the surface of infected cells and are released by the enzymatic activity of the viral neuraminidase (NA), which cleaves sialic acid from cell-surface glycoproteins and glycolipids. Examples of cellular restriction factors with known antiviral activity against influenza viruses are shown in orange.

Influenza viruses interact with host cell factors at every stage of their replication cycle and many studies have investigated the potential of specific host proteins to act as cellular restriction factors of influenza virus replication. Remarkably, for many restriction factors defined to date it is clear that influenza viruses have evolved strategies to circumvent or limit their impact. In this review, we will discuss restriction factors that have been shown to target different steps in the influenza virus life-cycle, including virus entry, replication and exit, and consider the viral countermeasures that are known to limit their impact. An overview of influenza virus replication and associated restriction factors is shown in Figure 2.

## 5. Restriction Factors That Target IAV Entry

Members of the IFITM family are amongst the most widely studied cellular restriction factors that act against influenza viruses. In humans, the family is composed of *ifitm1*, *ifitm2*, *ifitm3*, *ifitm5* and *ifitm10* genes with IFITM1, 2 and 3 reported to mediate antiviral activity against influenza and other viruses [29,30]. While the subcellular distribution of these molecules can vary, cumulative evidence indicates that IFITM1 is localised predominantly at the plasma membrane, while IFITM2 and 3 localise to endosomal and lysosomal compartments (reviewed in [31]). The antiviral activities of IFITM3 have been particularly well studied. IFITM3 does not inhibit influenza virus attachment or internalisation but instead traps virions in endocytic compartments, resulting in their degradation [30,32]. IFITM3 is also active against many other viruses that infect cells via endocytic pathways. The mechanism by which IFITM3 inhibits infection is not fully understood, although current evidence indicates that it can modulate endosomal membranes to block virus-mediated fusion. IFITM3 may block the formation of fusion pores after virus-endosome hemifusion has occurred [33], with recent evidence implicating the importance of an amphipathic helix for IFITM3-dependent inhibition of influenza virus entry [34]. IFITM3 has also been implicated in protection against IAV infections in vivo and IAV infection of IFITM3 knockout mice was associated with exacerbated disease and mortality [32,35]. Moreover, previous reports have identified an enrichment of the rs12252-C allele of IFITM3 in patients hospitalised during the H1N1 IAV pandemic in 2009 [35] and the correlation between this allele and influenza severity has been validated in some, but not all, subsequent human cohort studies. Currently, the role of other IFITM-family proteins (e.g., IFITM-1/2) in recognition and response to influenza viruses is less clear.

In recent studies, zinc metallopeptidase STE24 (ZMPSTE24) was reported to inhibit a diverse range of viruses, all of which required access to endosomal compartments for virus entry [36]. Influenza virus infection of ZMPSTE24-deficient mice was also associated with increased viral titres, enhanced cytokine production and increased mortality [36]. Of interest, ZMPSTE24 was detected in IFITM protein complexes and restricted a similar spectrum of viruses to IFITM family proteins. Moreover, genetic complementation indicated that ZMPSTE24 is required for the antiviral activity of IFITMs. Thus, while both IFITMs and ZMPSTE24 have the potential to inhibit cytosolic entry of IAV and other viruses, further studies are required to elucidate the specific mechanisms by which virus entry is blocked.

Using CRISPR/Cas9 activation technology to perform a genome-wide overexpression screen, Heaton et al. identified a number of host proteins with anti-IAV activity that are likely to target attachment and entry stages of the IAV life-cycle [37]. For example, B4GALNT2 is a glycosyltransferase that causes the specific addition of a GalNAc residue to the sub-terminal galactose moiety of α2,3-linked sialic acid containing glycans and overexpression of B4GALNT2 inhibited cell-surface attachment of IAV strains with HA preference for α2,3-linked sialic acid. Moreover, the host proteins RIN2 and TM9SF2, which modulate endocytosis and endosomal maturation, respectively, were also implicated in blocking IAV infection [37] although the mechanisms by which they block infection are yet to be defined.

## 6. Restriction Factors That Interfere with Genomic Transcription and Replication

Most of the antiviral factors against influenza virus that have been identified to date target genomic replication and/or translation during the influenza virus life cycle. Certain restriction factors target particular viral proteins (e.g., NP, M1, polymerase basic (PB)1 and PB2), to promote their degradation and/or alter cellular localization, whilst other restriction factors target the viral genome itself. While virus attachment, entry and fusion are generally not altered by expression of these restriction factors, impaired viral replication will also ultimately inhibit virus release.

### 6.1. Mx Proteins and Other GTPases

The dynamin superfamily of GTPases share similar structural and biochemical properties including a N-terminal GTPase domain, a middle domain (which can be associated with self-oligomerization) and a C-terminal GTPase effector domain [38]. It includes IFN-inducible GTPases such as Mx proteins, the very large-inducible GTPases, p47 immunity-related GTPase (IRG) and guanylate-binding proteins (GBPs) [39]. Mx proteins are expressed in virtually all vertebrates and their antiviral activities have been intensely studied. Different Mx proteins associate with particular intracellular compartments in the cytoplasm and nucleus. In humans, cytoplasmic Mx1 (usually known as MxA) is able to inhibit a broad spectrum of viruses whereas the related Mx2 (usually known as MxB) localizes to the nuclear envelope and acts as a restriction factor for HIV-1 and other primate lentiviruses (reviewed in [40]). In rodents, Mx1 and Mx2 localise to the nucleus and cytoplasm, respectively, and this correlates with their antiviral profile; Mx1 inhibits influenza and other viruses that replicate in the nucleus whereas Mx2 inhibits viruses that replicate in the cytoplasm. While most inbred laboratory mouse strains lack Mx1, transgenic expression of murine Mx1 or the human homolog called MxA, results in high levels of resistance to IAV infections [41,42], although mutations in the viral NP can facilitate escape from MxA restriction in vivo [41].

Both human MxA and mouse Mx1 proteins display potent antiviral activity against influenza viruses by targeting vRNPs. Human MxA blocks at least two steps in the influenza virus life cycle. Firstly, incoming vRNPs are retained in the cytoplasm [43,44] although it is currently unclear if this requires IFN-induced auxillary molecules that act in conjunction with MxA as suggested by some [44], but not other studies [43,45]. Secondly, the amplification of vRNA from cRNA copies (secondary transcription) is also blocked, possibly via cytoplasmic sequestering of newly synthesized NP and PB2, but the exact mode of action is not yet defined [46,47]. In contrast, the nuclear murine Mx1 targets vRNP-associated viral polymerase activity to block primary transcription of viral genes [46,47]. While Mx proteins are known form dimers, tetramers and higher oligomeric ring-like structures, the molecular mechanisms by which they mediate antiviral activity are still not clear, although MxA does require GTP hydrolysis and oligomerization for antiviral activity. The variable sensitivity of different strains of IAV to Mx proteins is determined by the viral NP, the main structural component of vRNPs [47]. Of note, virus strains of avian origin were more sensitive to the effects of Mx1 and MxA than strains of human origin, suggesting that MxA might represent an important barrier against introduction of avian IAV into the human population. The viral PB2 may also represent a target for mouse Mx1, since its overexpression can outcompete the activity of Mx1 [48,49]. Note that human MxB and murine Mx2 do not appear to be potent inhibitors of influenza virus replication.

Guanylate-binding proteins (GBPs) represent an additional group of IFN-inducible GTPases. To date, 11 GBPs have been described in mice and 7 in humans, and many studies have defined roles for GBPs in cell-autonomous immunity against intracellular pathogens, in particular against intracellular bacteria and protozoa (reviewed in [50]). While much less is currently known regarding antiviral activity, human GBP1 mediates anti-IAV activity [51]. Functional GTPase activity was essential for the inhibition of IAV replication and the viral NS1 bound to GBP1 to antagonise both GTPase and anti-IAV activities [51]. In contrast, GTP binding, but not hydrolysis, was essential for the ability of a splice variant of human GBP3 to repress the activity of the viral polymerase complex and inhibit IAV replication [52]. A recent study also demonstrated that overexpression of human GBP5 inhibited virus replication by enhancing the expression of virus-induced IFN and IFN-related effectors [53]. These findings highlight the potential of GBPs as anti-IAV effectors although further studies are required to assess the antiviral activities of different GBP-family members, as well as their relevance in vivo.

### 6.2. Protein Kinase R

Protein kinase R (PKR) is an IFN-inducible protein kinase that is activated on binding to dsRNA and displays antiviral activity against a broad range of viruses. Once activated, PKR phosphorylates itself and downstream substrates, including the eukaryotic initiation factor 2α-subunit (eIF-2α) and IκB (reviewed in [54]). Phosphorylation of eIF2α results in potent inhibition of viral protein synthesis, thereby limiting viral replication. In addition, PKR phosphorylates IκB to promote the activation nuclear factor-kappa B (NF-κB), which positively regulates the transcription of IFN genes and contributes to the expression of IFN-stimulated genes. Factors such as the virus strain, infectious dose and genetic strain of the host, may be relevant to reports indicating that mice lacking PKR do [55], or do not [56] show increased susceptibility and antiviral responses to influenza virus infection.

Influenza viruses employ multiple strategies to inhibit the antiviral actions of PKR. For example, IAV infection results in posttranslational activation of the cellular protein p58IPK which can bind to PKR and inhibit its dimerization and phosphorylation [57]. Although wild-type IAV and IBV do not activate PKR efficiently, mutant viruses with defects in the NS1 gene (A/NS1 and B/NS1, respectively) are potent PKR activators [58,59,60]. Consistent with these findings, viral NS1 proteins have been shown to repress PKR activity [61,62]. Currently, the precise mechanisms underlying NS1-mediated inhibition of PKR activity are not fully understood, but may involve sequestration of viral RNA [62] and/or upregulation of vault RNAs through NS1 to impair PKR-dependent antiviral responses [63].

### 6.3. OAS-Family Proteins

The 2′,5′-oligoadenylate (2-5A) synthetases (OAS) are a family of ISGs characterized by their ability to synthesize 2-5A, which induces RNA degradation by activating RNaseL. In humans, binding of OAS1, 2 and 3 to dsRNA results in activation of enzymatic activity to synthesise 2-5A from ATP. OAS-generated 2-5A binds to cytoplasmic RNaseL to trigger its dimerization and activation (reviewed in [64]). Activated RNaseL then degrades viral and cellular ssRNAs, thereby limiting virus replication and inducing apoptosis of infected cells. In addition, some of these degradation products have been reported to bind to and activate RIG-I, and therefore serve to enhance IFN production [65]. While OAS1, 2 and 3 bind dsRNA and can synthesise 2-5A, OAS3 displayed a higher affinity for dsRNA and knockout of OAS3, but not OAS1 or OAS2, resulted in reduced RNAseL activation and enhanced IAV replication [66]. The NS1 protein of IAV contains an N-terminal RNA binding domain which binds to and sequesters dsRNA resulting in inhibition of the OAS/RNaseL system as well as inhibition of dsRNA-dependent signaling required for new IFN production [67]. Human oligoadenylate synthetase-like (OASL) does not harbor the catalytic activity required for synthesizing 2-5As and differs from the other human OAS family members by having two C-terminal ubiquitin-like domains. OASL binds to RIG-I and mimics polyubiquitin, thereby enhancing the sensitivity of RIG-I to activation and, in turn, leading to enhanced IFN induction following infection with certain RNA viruses [68]. Note that the role of OASL during IAV infection is yet to be formally addressed.

### 6.4. Other Restriction Factors That Interact Directly with Viral RNA

IFIT (IFN-induced protein with tetratricopeptide repeats (TPRs))-family proteins contain four members in humans, namely IFIT1 (ISG56), IFIT2 (ISG54), IFIT3 (ISG60) and IFIT5 (ISG58), and are induced to high levels in response to IFN signaling and/or viral infections [69]. While it is clear that IFIT proteins mediate antiviral activity against a broad range of viruses via suppression of translation initiation, binding of uncapped or incompletely capped vRNA and/or sequestering viral proteins or RNA in the cytoplasm (reviewed in [31]), their role during influenza virus infections remains to be clarified. IFIT1 bound directly to viral RNA in IAV-infected cells and silencing of IFIT1, IFIT2 and IFIT3 was associated with enhanced IAV replication, however ectopic expression of individual IFITs did not impair virus replication [70]. These findings suggest that the entire IFIT complex may be required for effective inhibition of IAV. However, more recent in vitro (deletion and overexpression in human and murine cells) and in vivo (mouse model) studies indicate that IFIT1 is not a dominant restriction factor against influenza viruses, likely due to the low binding affinity of IFIT1 for 5′-ppp RNA [71].

Adenosine deaminase acting on RNA (ADAR)1 is a RNA editing enzyme that modifies cellular and viral RNAs, including coding and noncoding RNAs. IFN-induced ADAR1 is highly expressed in lung cells and has been reported to act as a restriction factor against paramyxoviruses and orthomyxoviruses, including IAV [72]. However, ADAR1 has also been reported to act as a proviral factor promoting replication of many viruses, including IAV [73]. Thus, further studies are required to clarify the role of ADAR1 in promoting or inhibiting IAV replication in vitro and in vivo.

### 6.5. Restriction Factors That Target Viral Proteins

Different restriction factors target specific viral proteins, but all ultimately modulate replication of the viral genome and/or later stages in the virus life cycle. For example, Moloney leukemia virus 10 (MOV10) is an IFN-inducible host protein that binds to the viral NP to inhibit its interaction with importin-α. This results in retention of NP in the cytoplasm and subsequent inhibition of the vRNP complex [74]. DDX21 RNA helicase acts to inhibit assembly of the viral RNA complex by binding the viral PB1, resulting in reduced synthesis of viral RNA and proteins. [75]. However, DDX21-mediated anti-IAV activity is countered by the viral NS1 protein which binds to DDX21 and displaces PB1 from DDX21, suggesting that sequential interactions with different IAV proteins may transform the DDX21 host restriction factor into a host regulator of viral gene expression. Recent studies demonstrate that Plakophilin 2 (PKP2) represents an additional host factor that competes with PB2 for binding to the viral PB1, thereby inhibiting the activity of the viral polymerase and subsequent viral replication [76]. The human IFN-induced gene *ISG20* encodes a 3′ to 5′ exonuclease with specificity for single-stranded RNA and, to a lesser extent, for DNA. Initial studies demonstrated that stable expression of ISG20 in HeLa cells reduced yields of IAV and other viruses [77] and recently Qu et al. confirmed that ISG20 expression was associated with suppression of the viral polymerase and that exonuclease activity was essential for its anti-IAV activity [78]. While MOV10 binds to the viral NP to prevent its import into the nucleus, binding of ISG20 to NP this does not alter its cellular location [74]. It is currently unclear if ISG20 binds directly to NP or perhaps to vRNA associated with NP given its high affinity for RNA substrates.

Cyclophilin A (CypA), a member of the immunophilin superfamily with peptidyl-prolyl cis-trans isomerase activity, is a predominantly cytoplasmic protein that displays chaperone-like activity and assists in protein folding [79]. While CypA promotes replication of certain viruses, it can suppress replication of others, including IAV. In early studies, CypA was shown to interact with the M1 protein of IAV to suppress viral replication [80]. Subsequent studies indicated that CypA did not affect replication of the IAV genome or nuclear export of viral mRNAs. Instead, it appeared to regulate synthesis of the M1 protein and/or accelerate degradation of M1 by the ubiquitin proteasome system 2 [81], although further studies are required to elucidate the exact mechanisms by which CypA mediates anti-IAV activity. Transgenic mice overexpressing CypA showed reduced susceptibility to IAV infection and this was associated with reduced virus replication and pathological lesions in the lung [82]. Of interest, Cyclophilin E (CypE) mediates anti-IAV activity via distinct mechanisms, binding to viral NP to interfere with NP self-association, as well as with NP-PB1 and NP-PB2 interactions, resulting in inhibition of viral replication and transcription [83].

Zinc finger antiviral protein (ZAP) is an IFN-inducible host factor with two isoforms that arise from alternative splicing, giving rise to long (ZAPL) and short (ZAPS) isoforms that differ only at the C-termini [84]. The C-terminal poly(ADP-ribose) polymerase (PARP) domain of ZAPL binds the PA and PB2 polymerase proteins, resulting in proteasomal degradation. The viral PB1 protein counteracts ZAPL antiviral activity by binding to an adjacent region of ZAPL, causing PA and PB2 to dissociate and thus escape degradation [85]. Recent studies demonstrated that ZAPS, which lacks the PARP domain, inhibits the expression of the IAV proteins PA, PB2, and NA by reducing viral mRNA levels and repressing its translation. Moreover, the antiviral activity of ZAPS was antagonized by the viral NS1 [86]. Thus, ZAP uses two distinct mechanisms to inhibit IAV replication and these are antagonised by different viral proteins suggesting that ZAP proteins play an important role in restriction of IAV infection.

ISG15 is an IFN-induced ubiquitin-like protein that binds covalently to target proteins via the sequential action of three enzymes that are also induced by type I IFNs: the E1 activating enzyme UbE1L; the E2 conjugating enzyme UbcH8; and the major E3 ligase Herc5 (reviewed in [87]). In the context of IAV infection, the NS1 protein represents the predominant target with limited ISG15 modification of other viral proteins reported [88]. Conjugation of ISG15 to the NS1 protein results in loss of one or more of its functions, thereby inhibiting virus replication. Although transfection experiments indicated that multiple lysine residues within NS1 can be modified by ISG conjugation, in virus-infected cells ISG15 modification occurred predominantly at only one or two lysine residues [88,89]. Of interest, the NS1 protein of IBV binds to ISG15 and inhibits its conjugation to target proteins whereas the NS1 of IAV does not bind ISG15 [90]. Moreover, the NS1 protein of IBV has been reported to bind only human and non-human primate ISG15 [91]. ISG15-deficient mice show increased susceptibility to infections with strains of IAV and IBV [92], consistent with an important antiviral role in vivo.

Tripartite motif (TRIM) proteins are key components of the host response to viral infections, functioning as direct antiviral restriction factors or modulating signaling cascades that lead to proinflammatory cytokine induction. There are now more than 80 known TRIM-family proteins in humans and most are E3 ubiquitin ligases. TRIM proteins are characterized by an N-terminal Really Interesting New Gene (RING) finger domain, one or two B-boxes and a coiled-coil. Additional C-terminal domains have been used to further classify TRIM proteins. Of the TRIMs implicated in regulation of innate immunity, a number can modulate PRR-mediated signaling cascades elicited in response to virus infection, as well as modulating IAV-induced autophagy and autophagy-mediated antiviral defences (reviewed in [93]). Other TRIMs have been reported to act as restriction factors for different viruses, including IAV. For example, the anti-IAV activity of TRIM22 was mediated by E3 ligase-dependent polyubiquitination of the viral NP, thereby promoting proteosomal-dependent degradation [94]. Similarly, TRIM32 binds to and ubiquitinates the viral PB1, targeting it for proteosomal degradation, thereby inhibiting viral polymerase activity [95]. While TRIM22 expression was upregulated by type I IFN and by IAV infection, these treatments did not modulate TRIM32 expression. In contrast to TRIM22 and TRIM32, the ability of TRIM56 to inhibit replication of IAV and IBV occurred independently of E3 ligase activity [96]. TRIM56 localized to the nucleus of virus-infected cells, where its C-terminal tail was implicated in suppressing viral RNA synthesis. It is currently unclear if TRIM56 binds directly to viral RNA, viral proteins or perhaps even proviral host factors.

In addition to human MxA, recent studies suggest that Tu elongation factor, mitochondrial (TUFM) might represent an additional factor that limits interspecies transmission of avian IAV to humans. Generally, avian IAV do not replicate efficiently in human cells but a E627K substitution in the PB2 can overcome this restriction. Compared to human-signature PB2_627_K, avian-signature PB2_627_E showed higher binding affinity to TUFM in mitochondria and TUFM-dependent inhibition of viruses bearing PB2_262_E correlated with the induction of TUFM-dependent autophagy in infected human cells [97].

## 7. Blocking Virus Assembly and Egress

A number of cellular restriction factors that inhibit the late stages of influenza virus infection have also been identified. For example, cyclin D3 is a key regulator of cell cycle G0/G1 phase progression, however its ability to inhibit IAV release from infected cells occurred independently of its role in the cell cycle [98]. Overexpression studies confirmed that cyclin D3 did not impair viral protein synthesis but instead bound directly to the viral M2, thereby interfering with M1–M2 binding which is essential for proper assembly of progeny virions. Therefore, cyclin D3 represents one of the few restriction factors described thus far that appears to interfere with IAV virion assembly.

Bone marrow stromal cell antigen (BST)-2 (tetherin, CD317, HM1.24) is an IFN-induced protein implicated in inhibiting the release of IAV and other viruses from the surface of infected cells (reviewed in [99]). However, while some studies reported that BST-2 could inhibit release of influenza virus-like particles (VLPs) [100,101] and infectious IAV [102,103,104,105,106] from cells, other studies dispute this [100,107,108,109]. Recent studies support a role for BST-2 in the restriction of IAV although sensitivity appeared to be strain specific [101,105]. The M2 protein of IAV binds to BST-2 to limit its anti-IAV activity [110] and the viral NA can also antagonise BST-2, possibly via enzymatic desialylation which in turn affects BST-2 maturation [101,102,103]. The effectiveness of these viral antagonists in different experimental systems (e.g., VLPs versus infected cells) and between virus strains may well be relevant to discrepancies between studies. Of interest, mouse models indicate that endogenous BST-2 does not play a major protective role following infection with either IAV [108] or IBV [111], nor was endogenous BST-2 a major factor restricting IAV entry into or release from primary AEC or MΦ [108].

Viperin (or virus inhibitory protein, endoplasmic reticulum-associated, interferon inducible), has been reported to inhibit the replication of different viruses by a variety of mechanisms (reviewed in [112]). In the case of IAV, viperin appears to act late in the virus life cycle, inhibiting virion release from infected cells by reducing the biosynthesis of isoprenoids, thereby affecting lipid raft formation [113]. While additional studies confirmed that viperin could restrict IAV budding in vitro [103,114], viperin-deficient mice and control animals did not show significant differences in viral load or pulmonary lung damage following IAV infection [114], arguing that endogenous viperin may not function as a major restriction factor against IAV.

Following release of influenza virus from infected cells, cleavage of the viral HA glycoprotein is critical to allow subsequent infection of target cells. Plasminogen activator inhibitor 1 (PAI-1) is an ISG that can restrict IAV spread via its ability to inhibit airway proteases required for the extracellular cleavage and therefore maturation of the viral HA glycoprotein [104] Further, partial PAI-1 deficiency, attributable to a polymorphism in human *SERPINE1*, conferred increased susceptibility to IAV in vitro. Thus, PAI-1 represents the first ISG to be described which mediates direct antiviral activity in the extracellular environment.

## 8. Conclusions and Perspectives

As discussed above, diverse families of host proteins have been implicated as restriction factors that inhibit IAV replication. Many target conserved features of virus replication such as virus entry (IFITM3), genomic replication (OAS/RNaseL), protein translation (PKR) and virus egress (BST-2) and therefore tend to display antiviral activity against many different viruses. Despite this, the function of most of the hundreds of ISGs induced following influenza infection still remain unknown. Certainly, some well-established restriction factors that act against other viruses (e.g., apolipoprotein B mRNA editing enzyme, catalytic polypeptide-like (APOBEC)3G-family proteins) are induced, but do not function as major restriction factors against influenza viruses [115]. Of the restriction factors identified to date, the mechanisms underlying their anti-IAV activity are still poorly defined. In fact, mechanistic details are still lacking for even some of the most intensely studied ISGs such as Mx and IFITM3. Moving forward, defining mechanisms underlying the ability of different restriction factors to inhibit influenza viruses is critical to define their role in antiviral immunity and to assess their potential as targets for the development of novel antiviral treatments.

RNAi screens designed to identify host cell factors essential for influenza virus replication (and hence potential targets for novel antiviral drugs) have also identified host restriction factors with antiviral activity. These and other genome-wide analyses, in combination with ectopic overexpression and/or gene silencing studies, have accelerated the identification of antiviral restriction factors. However, a number of constraints should be considered when using in vitro approaches to assess putative restriction factors. For example, ectopic overexpression can alter cellular distribution and hence the antiviral activity of particular restriction factors. Species compatibility can also be an important issue if particular restriction factors work in concert with additional cellular proteins that may differ between species. In addition, the use of cells relevant to infection by the virus of interest, as well the levels of endogenous restriction factors expressed in distinct cell types, should also be considered. In fact, the expression of particular restriction factors may well be important in determining the permissiveness of different cell types to infection. For example, pulmonary endothelial cells, but not AEC, express high constitutive levels of IFITM3 that contribute to blocking human IAV infection although some avian IAV escape this restriction, consistent with the tropism of avian but not human IAV for endothelial cells [116]. While most studies have focused on identification of restriction factors that limit IAV infection in epithelial cells and other cell lines, whether MΦ and DC express distinct restriction factors that contribute to their ability to block productive replication by seasonal IAV remains to be determined.

Despite induction of multiple restriction factors with known antiviral activity, influenza virus infections often cause disease, consistent with expression of virus-encoded antagonists (as discussed above) and/or inefficient inhibition of IAV by endogenous restriction factors. It is possible that influenza viruses may also exploit particular restriction factors as has been described for other viruses. For example, upregulation of BST-2 or viperin by human cytomegalovirus (HCMV) has been associated with enhanced virus entry and infection [117,118]. While some well-studied restriction factors mediate potent anti-IAV activity others are more modest inhibitors, particularly at the levels expressed in AEC and other relevant cell types. Understanding the combined effects of restriction factors and their contribution to innate immunity to IAV in vitro and in vivo represents an important avenue for future research.

Compared to influenza viruses, much less is known about cellular restriction factors that block the replication of other respiratory viruses, including respiratory syncytial virus (RSV), parainfluenza viruses (PIV) and human metapneumovirus. ISG15 [119], viperin [120] and IFITM1/3 [121] have all been reported to act against RSV. A recent study also defined a number of ISGs with antiviral activity against PIV-3, including indoleamine 2,3-dioxygenase (IDO), IFIT1, IFITM1 and PKR [122]. To our knowledge, the antiviral activity of specific cellular restriction factors against HMPV are yet to be reported. A growing body of evidence highlights the potential of therapeutics targeting cellular proteins, including antiviral agents in clinical trials and/or approved for use [123]. Thus, the identification and characterisation of specific restriction factors that act against a wide range of respiratory viruses provides a rationale for targeting specific molecules as candidates to provide broad spectrum protection against respiratory virus disease.
